# Association between circadian rhythm and sleep quality among nursing interns: A latent profile and moderation analysis

**DOI:** 10.3389/fnins.2022.995775

**Published:** 2022-11-03

**Authors:** Xiaona Wu, Yingzi Lu, Xian Xie, Rongjie Chen, Ningning Zhang, Chen Zhou, Zengjie Ye

**Affiliations:** ^1^School of Nursing, Guangzhou University of Chinese Medicine, Guangzhou, China; ^2^School of Nursing, Southern Medical University, Guangzhou, China; ^3^School of Nursing, Guangdong Pharmaceutical University, Guangzhou, China

**Keywords:** perceived stress, circadian rhythm, sleep quality, latent profile analysis, moderation analysis, Be Resilient to Nursing Career (BRNC), nursing interns

## Abstract

**Background:**

Disturbances in circadian rhythms are common among night-shift workers and result in poor sleep quality. Nevertheless, the heterogeneity of circadian rhythms and their relationship with sleep quality is less explored in nursing interns. Therefore, we aimed to identify the latent subtypes of circadian rhythm, explore their relationship with sleep quality, and evaluate their moderating role between perceived stress and sleep quality in nursing interns.

**Materials and methods:**

In all, 452 nursing interns were recruited between October 2020 and January 2021 from Be Resilient to Nursing Career (BRNC), which is a multicenter, prospective cohort of a career growth program for nursing students. They were assessed using the 10-item Chinese Perceived Stress Scale, Circadian Type Inventory, and Pittsburgh Sleep Quality Index. Latent profile analysis and moderation analysis were performed.

**Results:**

Overall, 72.3% of the nursing interns reported poor sleep quality. We identified three latent subtypes of circadian rhythms, namely, Vigorousness (40.1%), Inadaptability (18.6%), and Flexibility (41.1%). Females (OR = 1.97, 95% Cl: 1.01–3.83, *P* = 0.047) with normal body mass index (OR = 1.62, 95% CI: 0.95–2.76, *P* = 0.078) were prone to Flexibility. Circadian rhythm types significantly moderated the association between perceived stress and sleep quality (*P* < 0.05).

**Conclusion:**

Nursing interns suffer from poor sleep. There exists heterogeneity of circadian rhythm subtypes in nursing interns, and attention should be paid to those with Inadaptability type. The association between perceived stress and sleep quality is significantly moderated by circadian rhythm subtypes.

## Introduction

Circadian rhythms refer to the behavioral and metabolic changes regulated by the circadian clock, which maintains synchronized changes with the natural light–dark cycle (Hastings et al., 2007; Zare et al., 2017). Sleep quality consists of sleep latency, number of awakenings after falling asleep, and sleep efficiency (Facco et al., 2017), which are associated with circadian rhythm (Zare et al., 2017). A recent study indicated that occupational stress might enhance the risk of poor sleep quality by the gene polymorphism of period circadian regulator 3 (*PER3*) gene (Peng et al., 2022). In a recent study, approximately 43.2% and 63.2% of Chinese night-shift workers had abdominal obesity and poor sleep quality, respectively (Sun et al., 2018). In addition, a large survey indicated that approximately 24.4–32.4% of night-shift workers reported sleep disorders (i.e., insomnia, excessive drowsiness, sleepiness, etc.) (Flo et al., 2012; Chen et al., 2020). Thus, circadian rhythm misalignment and poor sleep quality may be the two most common health-related problems in the night-shift populations (Fido and Ghali, 2008).

The circadian rhythm is defined by three key parameters: the period, the phase, and the amplitude (Zhang and Jain, 2021). The normal rhythm may be disrupted by a change in period, phase, amplitude, or any combination of these through human activities or environmental factors such as night shifts and occupational stressors (Pallesen et al., 2021; Zhang and Jain, 2021). Due to the recognition of individual differences in human biology and their potential role in the adjustment to night work, several self-report measures have been developed that indirectly assess the characteristics of circadian rhythms (Di Milia et al., 2005). Early self-report measures focused on assessing differences in the circadian phase. According to these measures, activity preferences are correlated with circadian rhythm phase differences (Di Milia et al., 2005). Later on, Folkard et al. (1979) developed the Circadian Type Questionnaire (CTQ) based on three characteristics of circadian rhythms. In order to improve the predictive power of the scale for shift tolerance, the Circadian Type Inventory (CTI) was developed, based on the CTQ (Di Milia et al., 2004). The CTI is reported to possess strong psychological properties in shift work studies (Di Milia et al., 2005; Jafari Roodbandi et al., 2015; Chen et al., 2020).

**FIGURE 1:**
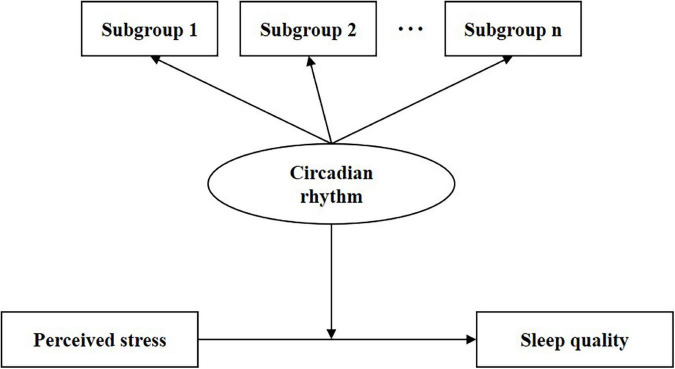
The hypothetical framework for circadian rhythms and sleep quality in nursing interns.

Night-shift work was developed in hospitals to ensure high-quality care; however, it leads to circadian misalignment and poor sleep quality among nurses (Di Muzio et al., 2021). The internship period is the duration for interns to acclimate to night-shift work. Nursing interns are a special subgroup of the nursing team and have received tremendous attention from multidisciplinary researchers (Mei et al., 2022c). Nursing interns experience transition-related stressors as well as non-habitual sleep–wake schedule (Zhang et al., 2019), which leads to night-shift intolerance and poor sleep quality among them (Huang et al., 2021). Previous studies reported that approximately 22.8–38.2% of nursing interns experience sleeping problems (Lai et al., 2020; Gao et al., 2021). Additionally, circadian rhythm disruption affects the levels of ghrelin, leptin, insulin, cortisol, and melatonin among night-shift workers, causing poor sleep quality (Ulhôa et al., 2015; Huang et al., 2021). Poor sleep adversely affects their physical and mental health (e.g., cardiovascular risk, mood disorders, etc.) as well as their work performance (Chang and Li, 2019). Therefore, it is important to pay attention to and address the sleep problems of nursing interns. Nonetheless, the relationship between circadian rhythm and sleep quality is less explored among nursing interns. In addition, there exists heterogeneity in circadian rhythms, and individuals with different latent profiles of circadian rhythms exhibit different responses to night-shift work (Axelsson et al., 2004; Boivin and Boudreau, 2014). However, traditional statistical methods are difficult to explore the psychological complexity of circadian rhythms especially when heterogeneous subgroups exist, and more advanced techniques should be performed in the present study (Choi et al., 2019). The Latent Profile Analysis (LPA) as a person-centered approach could more accurately identify heterogeneity within a population than the variable-centered approach (Kongsted and Nielsen, 2017). Hence, the current study was designed to meet the following objectives: (1) Identify latent subgroups with different circadian rhythm types by Latent Profile Analysis (LPA); (2) Compare sleep quality among subgroups with LPA-based circadian rhythm types; (3) Evaluate the moderating role of LPA-based circadian rhythm types between perceived stress and sleep quality. The hypothesized framework is described in Figure 1.

## Materials and methods

### Participants and procedure

We conducted a multicenter, cross-sectional survey of nursing interns in Guangzhou City, China, between October 2020 and January 2021. In all, 478 nursing interns were recruited from Be Resilient to Nursing Career (BRNC) by cluster sampling (Chen et al., 2022; Mei et al., 2022a,b,c; Wu et al., 2022). Of these, 452 completed the survey resulting in a response rate of 94.6%. The minimum sample for LPA analysis with 80% accuracy is 300 (Swanson et al., 2012). Thus, the sample size of 452 in the current study was efficiently powerful. The inclusion criteria were as follows: (1) nursing interns who had been working on night shifts from the start of their internship until the survey month and (2) clinical internship ≥3 months. Additionally, participants with any mental disorder diagnosed by clinical psychiatrists in the last 6 months were excluded.

### Measures

#### Demographic characteristics

Demographic characteristics including gender and educational level were collected. Also, sleep-related characteristics, such as the habit of consuming caffeinated drinks, regularity of diet, and body mass index (BMI) were collected according to previous research (St-Onge et al., 2016; Sun et al., 2018; Gross et al., 2020; Ono et al., 2020; Ribas-Latre et al., 2021).

#### Measure of circadian rhythm

The Chinese version of the Circadian Type Inventory (CTI) assesses circadian flexibility and languidity in shift nurses and was validated by Qi et al. (2019). It has two domains including Flexible/Rigid (FR) and Languid/Vigorous (LV). The total score ranges from 11 to 55 with higher scores indicating a greater ability to handle with shift-related sleep problems. The cut-offs for FR and LV are 18.75 and 22.5, respectively (Di Milia et al., 2004; Jafari Roodbandi et al., 2015). In this study, Cronbach’s alpha was 0.879 for FR and 0.764 for LV.

#### Measure of sleep quality

Pittsburgh Sleep Quality Index (PSQI) assesses sleep quality and can be applied to patients with sleep disorders as well as the general population. It has seven domains including sleep quality, sleep latency, sleep duration, habitual sleep efficiency, sleep disturbances, use of sleep medications, and daytime dysfunction (Buysse et al., 1989). The cut-off for PSQI is 5 (Jafari Roodbandi et al., 2015). The Cronbach’s alpha was 0.852 in the current study.

#### Measure of perceived stress

The 10-item Chinese Perceived Stress Scale (CPSS-10) was developed by Cohen (1988), and the Chinese version is proven to be reliable (Ng, 2013). It assesses the perceived stress level of individuals in the last month. CPSS-10 has two domains including “Perceived Helplessness” and “Perceived Self-efficacy.” The total scores range from 0 to 40, with a higher score indicating a higher stress level. The cut-off for CPSS-10 is 26 (Cohen, 1988). In the present study, the Cronbach’s alpha was 0.877.

### Statistical analysis

First, demographic and sleep-related characteristics (categorical variables) were described as frequencies and proportions (%). Second, spearman correlation analysis was performed to assess the associations among perceived stress, circadian rhythm, and sleep quality. Strength of relationship was categorized as follows: weak (|r| < 0.3); moderate (0.3 ≤ | r| < 0.5); strong (|r| ≥ 0.5) (Cohen, 2013). In addition, Generalized Additive Model (GAM) was employed to estimate the non-linear associations (Hastie and Tibshirani, 2017). Third, LPA was performed to identify potential subgroups with different circadian rhythm types. It began with a one-class model, continuing until fit indices could not be significantly improved. Bayesian Information Criteria (BIC), Akaike’s Information Criteria (AIC), Lo-Mendell-Rubin (LMR), and Entropy value were utilized as main fitting indicators (Muthén and Muthén, 2017). Fourth, LPA-based differences in PSQI scores were estimated by Bayesian Factor. The Cauchy prior width was set at 0.7, which indicates a 70% probability of the actual effect size being between –0.5 and 0.5 (Molero Jurado et al., 2021). The Bayes factor value greater than 10 indicates strong relative evidence for a hypothesis (Quintana and Williams, 2018). Fifth, univariate (*P* < 0.2) and multivariate regressions were used to recognize potential indicators of LPA-based circadian rhythm types. Sixth, stratified analysis was performed to evaluate the difference in sleep quality among subgroups with different circadian rhythm types after controlling for potential covariates. At last, the moderating role of LPA-based circadian rhythm types (category variable) was estimated between perceived stress (continuous variable) and sleep quality (continuous variable). The data were run by Statistical Product and Service Solutions (SPSS, version 22.0), Mplus (version 8.3), Empower Stats (version 3.0), and JASP (version 0.16.0). Significance was set at 0.05.

## Results

### Demographic characteristics

A total of 452 nursing interns completed the survey (female, *N* = 380); 52.43% were accustomed to caffeinated drinks and 23.67% had irregular meal intake during the internship. The BMI of male and female interns were 20.36 (*SD* = 3.51) and 20.05 (*SD* = 3.35), respectively. The median of night shifts per month was three and 17.5% reported more than five per month. Other demographic details are presented in Table 1.

**TABLE 1:** Demographic characteristics and relevant variable differences in sleep quality.

Variables		*N*	Percentage (%)	*P*-value
Gender	Female	380	84.07	0.725
	Male	72	15.93	
The habit of drinking caffeinated drinks	No	215	47.57	0.719
	Yes	237	52.43	
Regularity of diet	Regular	345	76.33	0.003
	Irregular	107	23.67	
Night shifts (number per month)	≤2	224	49.56	0.073
	3∼4	149	32.97	
	≥5	79	17.48	
BMI	Normal	287	63.50	0.091
	Abnormal	165	36.50	
Perceived stress	Mild	410	90.71	0.419
	Severe	42	9.29	

BMI, body mass index; BMI “18.5∼23.9” was considered to normal, while BMI “<18.5, 24∼27.9, ≥ 28” were defined as abnormal according to Chinese standards.

### The analysis of the correlations among perceived stress, circadian rhythm, and sleep quality

**FIGURE 2:**
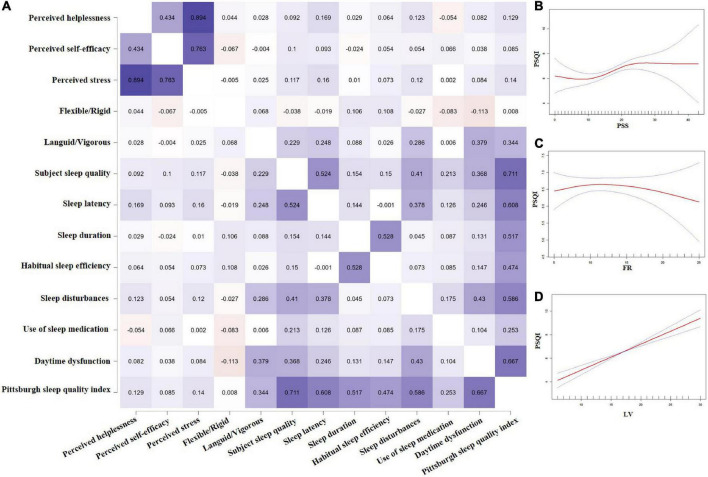
Spearman correlation Heatmap among perceived stress, circadian rhythms, and sleep quality **(A)**, and curve line regression of the above three variables **(B–D)**.

The mean and standard deviations of variables were perceived stress (16.91 ± 6.62), Flexible/Rigid (12.15 ± 4.68), Languid/Vigorous (17.11 ± 4.85), and PSQI (6.56 ± 3.08). Spearman correlations are described in Figure 2A. PSQI was positively associated with Languid/Vigorous (*r* = 0.34, *P* < 0.01) and perceived stress (*r* = 0.14, *P* < 0.05). The associations between PSQI, perceived stress, and Flexible/Rigid were non-linear (Figures 2B–D).

### Latent profiles analysis of circadian rhythm

**FIGURE 3:**
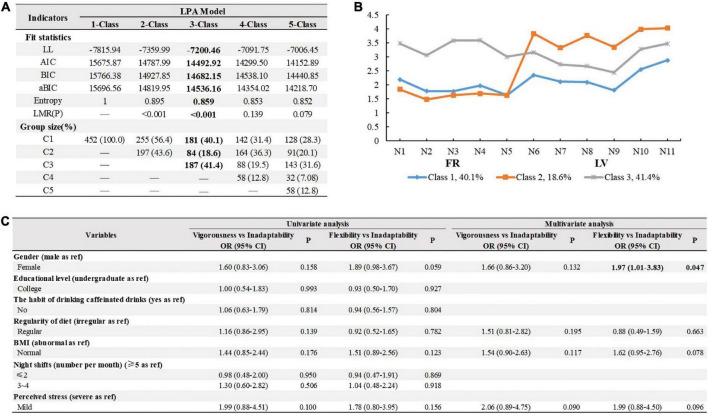
Fitting index and group size of latent profile analysis models and logistic regression results for predicting external features on the 3-class pattern. **(A)** Fitting index and group size of latent profile analysis models. Bold figures highlight the selected class solution. LL, Log-likelihood; AIC, Akaibe information criterion; BIC, Bayesian information criterion; aBIC, adjusted BIC; LMR, Lo, Mendell, and Rubin likelihood ratio test; C1, vigorous; C2, inadaptability; C3, flexibility. **(B)** Parameters for the final three-class patterns. FR, flexible/rigid; LV, languid/vigorous. **(C)** Univariate and multivariate logistic regression results for predicting external features on the 3-class pattern. BMI, body mass index; OR, Odds ratio; CI, confidence interval. Bold figures highlight statistically significant in the multivariate logistic regression.

One to five latent subgroups were checked based on fitting indicators and the three-class model was optimal in consideration of (1) relatively small AIC, BIC, and aBIC, (2) the sample size of each class was more than 50, and (3) a significant *P*-value of Lo-Mendell-Rubin (LMR). Other information is detailed in Figures 3A,B.

We named the latent profiles based on the overall distribution of the observed variables to distinguish heterogeneity in the circadian rhythms of interns. Three circadian rhythm types were identified: Vigorousness (40.1%, middle Flexible/Rigid-low Languid/Vigorous), Inadaptability (18.6%, low Flexible/Rigid-high Languid/Vigorous), and Flexibility (41.1%, high Flexible/Rigid-middle Languid/Vigorous). Logistic regression analysis showed that gender was the only indicator of circadian rhythm types (OR = 1.97, 95% CI: 1.01–3.83, *P* = 0.047), after controlling for the covariates (Figure 3C).

### Latent profile analysis-based differences in Pittsburgh sleep quality index scores

**FIGURE 4:**
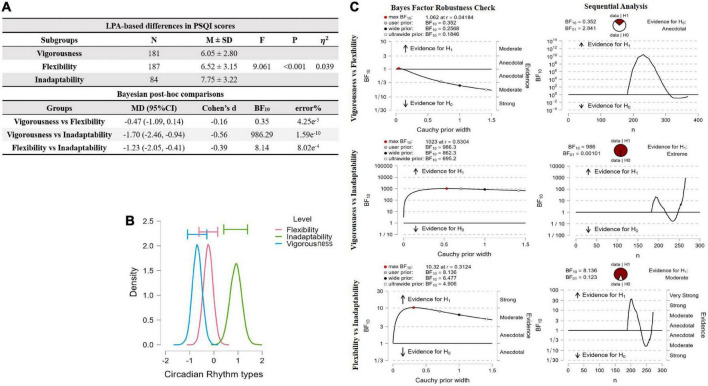
ANOVA comparison of PQSI scores across LPA-based groups and *post hoc* comparison by Bayesian factor analysis. **(A)** LPA-based differences on PSQI scores were estimated by Bayesian Factor. V, Vigorous group; F, flexibility group; I, inadaptability group; SD, standard deviation; MD, mean difference; BF, Bayes Factor; η^2^, eta squared represents the explained unique variance of a dependent variable by three homogenous subgroups. **(B)** ANOVA model averaged psterior distribution. **(C)** Inferential plots for Bayesian factor analysis.

The PSQI scores were significantly different between Vigorousness and Inadaptability (BF_10_ = 986.29, *d* = –0.56) and between Flexibility and Inadaptability (BF_10_ = 8.14, *d* = –0.39), but not between Vigorousness and Flexibility (BF_10_ = 0.35, *d* = –0.16). These findings were confirmed by Bayesian Factor Robustness analysis (Figure 4).

### Association between circadian rhythm types and sleep quality

**FIGURE 5:**
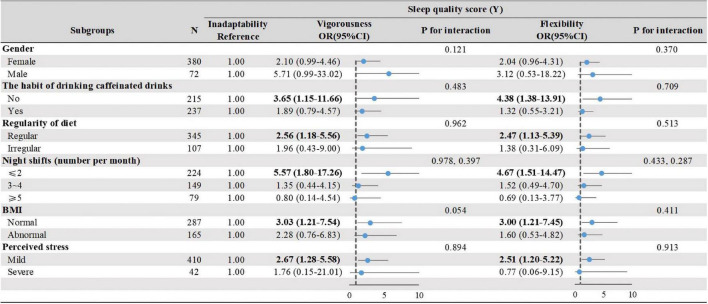
The effect of circadian rhythm subtypes on sleep quality by stratified analysis. Stratified associations between circadian rhythm types and sleep quality by physiological and environmental factors. Analyses were adjusted for covariates gander, the habit of drinking coffee, regularity of diet, night shifts, BMI, PSS when they were not the strata variables. OR, odds ratio; CI, confidence interval. Bold figures highlight statistically significant in the strata variables.

Compared with Inadaptability, Vigorousness and Flexibility generally had better sleep quality, especially in interns with no habit of drinking caffeinated drinks, regular diet, ≤2 night-shifts, normal BMI, and mild perceived stress although the *P*-values for interaction were all not significant (Figure 5).

### The moderating role of circadian rhythm types between perceived stress and sleep quality

**FIGURE 6:**
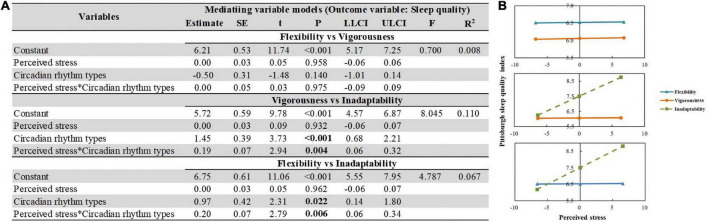
The moderating effect of circadian rhythm types between perceived stress and sleep quality. **(A)** Moderating role of circadian rhythm types in the association between perceived stress and sleep quantity. Regularity of diet was adjusted for each model. Independent variables were centered before analysis. **(B)** Simple slope plot of the interaction between circadian rhythm types and perceived stress on sleep quality.

The moderation analyses (Figure 6A) showed that the interactions of perceived stress and circadian rhythm types (Vigorousness vs. Inadaptability, Flexibility vs. Inadaptability) were associated with sleep quality (*B* = 0.20, *SE* = 0.07, *P* = 0.003, and *B* = 0.20, *SE* = 0.07, *P* = 0.004, respectively). However, no significant moderation effect was identified between Vigorousness and Flexibility groups at different levels of stress (*B* = 0.00, *SE* = 0.05, *P* = 0.939). Other information is listed in Figures 6A,B.

## Discussion

This study illustrated the significant impact of night-shift on the sleep quality of nursing interns. We found that 72.3% of the nursing interns did not receive adequate sleep, which is greater than that of nursing students in school (66.0%) (Marta et al., 2020) and the general Chinese population (25.0%) (Sun et al., 2015). According to Bjorvatn and Waage (2013), nurses who work in shifts are more likely to experience confusional arousal and nightmares than nurses who work only during the day. This phenomenon was hypothesized to be related to circadian misalignment and sleep deprivation (D’Ettorre et al., 2020), especially for fresh nurses (Ebrahimi et al., 2016).

This study was the first to examine latent profiles of circadian rhythm by LPA in a sample of nursing interns. Using the LPA, individuals could be categorized into groups share similarity but differ from individuals in other groups (Kongsted and Nielsen, 2017). According to the scoring patterns of the Circadian Type Inventory, they were named as Vigorousness (resilient to decreased sleep time), Inadaptability (vulnerable to sleep problems), and Flexibility (can well regulate unusual working hours). These results confirm the heterogeneity of circadian rhythms. Since the sleep–wake cycle of nursing interns with inadaptability (18.6%) was prone to disorders in the night-shift environment, they need more attention (Zare et al., 2017). In addition, the symptoms of sleepiness may be exacerbated by work fatigue, a reduction in alertness, and inability to recover from sleep (Boivin and Boudreau, 2014). Consistent with previous studies, we found that females were more prone to Flexibility and more resilient to disruptions in circadian rhythms compared with males (Fischer et al., 2017; Anderson and FitzGerald, 2020). The rhythms of estrogen and androgen receptors that act on the suprachiasmatic nucleus (SCN) are ascribed to sexual dimorphism, with females showing higher oscillation amplitudes and peaks for gene expression than males (Anderson and FitzGerald, 2020; Joye and Evans, 2021). The amplitude of the rhythm (i.e., the magnitude of the highest and lowest values of the periodic variation in the physiological system) is related to the adaptive adjustment of shift workers (Rajaratnam et al., 2013). In rodent experiments, female mice were more resilient to the destruction of genes and environment on circadian rhythm (Bixler et al., 2009).

We observed a significant difference in PSQI among three LPA-based circadian subgroups. Vigorousness and Flexibility groups, generally, had better sleep quality, especially in interns with no habit of drinking caffeinated drinks, a regular diet, ≤2 night shifts, normal BMI, and a mild level of perceived stress when compared with Inadaptability. These findings are in line with previous research (Muehlbach and Walsh, 1995; St-Onge et al., 2016; Ono et al., 2020; Ribas-Latre et al., 2021). A cross-sectional study involving 4,856 emergency nurses found that nurses who worked more than four night shifts per month suffered from poorer sleep compared with those who never worked in night shifts (Dong et al., 2020). This discrepancy in findings may be attributed to the age, as the mean age of the participants in this study was 21.19 years (*SD* = 0.85) compared to 35.30 years (*SD* = 6.80) in Dong et al. (2020). A study reported that sleep–wake circadian rhythms become disrupted with aging, resulting in more awakenings and night terrors, and less slow-wave sleep (Reilly et al., 1997). Nursing staff with higher age and length of service have lower sleep phase stability and rhythmic amplitudes, indicating a greater tolerance for night shifts than interns (Li et al., 2017; Jacobson and Hoyer, 2022). It should be noted that Vigorousness and Flexibility groups were not able to recover well from night shifts and had no better sleep quality than the Inadaptability group when the night shifts exceeded 2. This phenomenon could also be recognized in interns with severe stress levels.

This study confirmed that circadian rhythm types play significant moderating roles between perceived stress and sleep quality. When exposed to increasing stress, the Flexibility and Vigorousness groups exhibited stable sleep quality, while the Inadaptability group was prone to poor sleep quality. An observational and longitudinal study revealed that medical internship (like stress and night shifts) plays an important role in the worsening of sleep quality and mental health (Tafoya et al., 2019), which is consistent with this study. A random sample of 1,163 participants from Australia showed that both vigorous and flexible rhythms (low amplitude and non-rigid) were significantly more resilient, coped better, and required less daily sleep (Di Milia and Folkard, 2021). The importance of these findings lies in showing that Inadaptability group exhibits low levels of resilience and circadian adaptation, which is reflected in poor sleep quality. Additionally, individuals with high resilience tend to well regulate their emotions in response to stress and exhibit regular circadian cortisol rhythms (Chi et al., 2015). However, these confounders need further validation.

Some preventive interventions should be developed for the Inadaptability group. First, timely identification of interns with Inadaptability especially among those with severe stress and >2 night shifts per month. Second, a 60–90 min nap before a night shift is recommended, which is helpful for the body to complete a sleep cycle (Mednick et al., 2003). Third, moderate and timely intake of caffeinated beverages, such as coffee, tea, and functional drinks, can effectively enhance work alertness and efficiency. Studies have shown that 4 mg/kg is the appropriate intake ratio, but it should not be consumed at least 3 h before bedtime to avoid affecting subsequent rest (Muehlbach and Walsh, 1995). Lastly, Cognitive Behavioral Therapy for Insomnia (CBT-I) could also be recommended if necessary (Newsom, 2022).

### Limitations

Inevitably, the study has certain limitations. (1) The sample was collected from three universities in southeast China, which might not be representative resulting in selective bias. Thus, the findings of this study require further validation in a new sample with different backgrounds. (2) This study was cross-sectional in nature; hence, a causal relationship could not be determined, and a longitudinal study should be performed to replicate these findings. An ongoing 2-year follow-up assessment of this cohort (BRNC) will provide additional insights in the future. (3) The study included only questionnaire data for sleep, which does not match the objective observations. Hence, objective sleep data are required to validate these findings in future studies. Biological indicators, such as menstrual cycle, body temperature, melatonin, and cortisol, could be incorporated as objective calibrations of circadian rhythms, which will provide more insights into future research. (4) Several important confounders, including resilience (Liang et al., 2022; Mei et al., 2022a,c; Wu et al., 2022), and social support, could be considered in the mediation analysis resulting in improved fitting indicators.

## Conclusion

There is a high prevalence of poor sleep quality among nursing interns. The circadian rhythms of nursing interns are heterogeneous and attention should be paid to those with the Inadaptability type. Additionally, the circadian rhythm subtypes moderate the association between perceived stress and sleep quality. Reasonable shift schedules and decreasing occupational stress should be considered when exploring preventive measures for poor sleep among nursing interns.

## Data availability statement

The raw data supporting the conclusions of this article will be made available by the authors, without undue reservation.

## Ethics statement

The studies involving human participants were reviewed and approved by the Ethics Committee of The First Affiliated Hospital of Guangzhou University of Chinese Medicine [No: ZYYEC-ERK (2020) 132]. The patients/participants provided their written informed consent to participate in this study.

## Author contributions

XW: conceptualization, data curation, methodology, software, and writing—original draft. YL: investigation, resources, and validation. XX, RC, NZ, and CZ: investigation and resources. ZY: supervision and writing—review and editing. All authors contributed to the article and approved the submitted version.
